# Adolescent Volar Barton Fracture with Open Physis treated with Volar Plating using Buttressing Principle

**DOI:** 10.5704/MOJ.1507.014

**Published:** 2015-07

**Authors:** H Shah, V Chavali, R Daveshwar

**Affiliations:** Department of Orthopaedics, Government Medical College, Baroda, India

**Keywords:** volar Barton, Fracture, physis, buttress fixation

## Abstract

Objective: The objective of the study was to assess the outcome of Salter- Harris type III distal radius fracture fixed using the principle of buttressing and avoiding screw insertion through the physis.

Materials and Method: Eight school going children in the age group of 11-16 years with volar Barton fractures were treated with a volar plate using the buttress principle without inserting screws in the distal fragment. Patients were evaluated over a period of 18 months. Clinical evaluation was done using the Green O’Brien criteria and radiological evaluation using the Sarmiento criteria.

Results: The average union time was two months. All the patients had good to excellent functional outcome with full extension and flexion.

Conclusion: Buttress plating of volar Barton fractures in the adolescent age group is an excellent technique to achieve satisfactory outcome without violation of the physis.

## Introduction

The distal end of radius contributes to 75-80% of forearm growth and 40% of upper limb growth along with the ulna. The physis of the distal end of radius closes at the age of 17-18 years^1^.

The Volar Barton fracture of the distal end of radius is partially articular which requires anatomical reduction. Though Salter Harris type I and II injuries are common at the distal end of radius, type III and IV are rare^1^.

Intra articular malunion is of concern as there is greater risk of development of degenerative arthritis if the articular step off is more than 2mm^1^. A thorough search into the available literature regarding the management of volar Barton fractures in the adolescent age group revealed no specific information. We have applied the principle of buttress plating without fixation into the distal fragment in eight cases of Salter- Harris type III (partial articular) distal end radius fractures.

## Materials and Methods

We present eight cases of adolescent volar Barton fractures (Salter- Harris type III, AO type 23-B) in the age group of 11-16 years treated at our hospital between April 2011 and March 2013. Patients with an isolated fracture of the lower end of radius without concomitant injuries were included in this study.

After primary management at the emergency department, the patients identified for our study were subjected to surgery. Using a volar approach, fracture fragments were isolated and reduced by manipulation. A volar buttress plate was fixed using only proximal screws. A simple cortical screw immediately proximal to the fracture site was inserted first which reduced the articular segment. Fracture reduction was confirmed radiologically and the plate fixed further proximally, leaving the distal fragment free of any fixation.

Wrist mobilization to the extent permitted by pain and tolerance was started on the third post-operative day. After the first dressing, patients were discharged and followed up in the outpatient department. Sequential x-rays were taken, to assess fracture union. Clinical and radiological assessments were done using Green O Brien and Sarmiento scores respectively (Table I and II). Fracture union was clinically defined as no pain or tenderness during daily activity involving loading at the wrist joint and radiographically defined as trabeculae having bridged the main fracture fragment^2^.

**Table I:** Green and O’Brien score - clinical assessment

1	Pain (25 points)	
	None	25
	Mild	20
	Moderate	15
	severe	0
2	Range of Motion	
	(flexion+extension % of normal)	
	100%	25
	99-75%	15
	74-50%	10
	49-25%	5
	<25%	0
3	Grip Strength (% of normal)	
	100%	25
	99-75%	15
	74-50%	10
	49-25%	5
	<25%	0
4	Activities	
	Returned to regular employment	25
	Restricted Employment	20
	Able to work but unemployed	15
	Unable to work because of pain	0
5	Final result	
	Excellent	90-100
	Good	80-89
	Fair	65-70
	poor	<65

**Table II:** Sarmiento Assessment score- radiological criteria

excellent	Deformity	None
	Dorsal angulation	<=0
	Shortening	<3mm
	Loss of radial deviation	<4 degree
Good	Deformity	Slight
	Dorsal angulation	1-10 degree
	Shortening	3-6mm
	Loss of radial deviation	5-9 degree
Fair	Deformity	Moderate
	Dorsal angulation	11-14 degree
	Shortening	7-11mm
	Loss of radial deviation	10-14 degree
poor	Deformity	Severe
	Dorsal angulation	>15 degree
	Shortening	>12 degree
	Loss of radial deviation	>15 degree

## Results

Of the eight patients, seven were male and one female. All patients were in the 11-16 year age group. The mechanism of injury was common to all, being a fall on the outstretched hand following a road traffic accident. All patients were school going students at the time of trauma who presented themselves at the hospital on the same day of injury. Seven fractures were in the dominant upper limb. All fractures were closed. None had any neurological complications either due to fracture or due to the common surgical approach used.

A pre-bent T-plate was used in all patients. None had any infection post operatively and surgical wounds healed without complications.

The average time for union was 8 weeks, without any surgical complications. At final follow up, only one patient had mild wrist pain. Range of motion at wrist of all patients was between 90-100% compared to the opposite side. Grip strength measurement at final follow up was comparable to the opposite side. All returned to school within 3 months.

## Discussion

The peak incidence of all fractures of distal radius with open physes is at the adolescent growth spurt^1^. Nietosvaara et al also reported distal radius physeal injuries occuring at an average of 12 years in males and 11 years in females^3^. However, the volar Barton fracture in the adolescent age group is rare and therefore poorly reported in the literature. Open reduction is indicated for displaced Salter- Harris type III and IV fractures^1^. The one absolute indication for operative management in distal radius physeal injuries is a Salter- Harris type III or IV fracture which should be treated by anatomical reduction using the open technique^4^. Intraarticular distal end radius fracture requires anatomical reduction by open reduction and internal fixation, and the same is true for volar Barton injuries. In the rare displaced intra articular Salter- Harris type III or IV fractures, internal fixation should be without violation of the physis, as physeal arrest due to distal radius physeal injury is at least 2%^5^.

Adolescent volar Barton fractures are type IV Salter- Harris physeal injury which is a rare type of distal end radius fracture. Anatomical reduction and union should be the final outcome in the patients even with less than a year of growth remaining, as marked primary displacement and non anatomical reduction are independent risk factors for re-displacement, which results in poor outcome.

Although the volar Barton fracture with open physis can be managed by conservative means, using external fixation and Kirschner wires, only open reduction and plate fixation is recommended for anatomical reduction. Marco Rizzo *et al* reported that open reduction and plate fixation is a better treatment modality for the distal articular radius fracture than Kirschner wire and external fixation^6^. As adolescent distal radius involves the physis, plate insertion with screw fixation should avoid the physis to prevent growth disturbance.

Volar buttress plate fixation without insertion of screws in the distal fragment is an excellent technique which provides anatomical reduction and excellent functional outcome in adolescent type III Salter-Harris (AO 23-B) distal radius physeal injuries. We recommend the study of a large group by this method to further justify the use of buttress plate in treatment of distal radius type III Salter-Harris fractures.

**Fig. 1:**
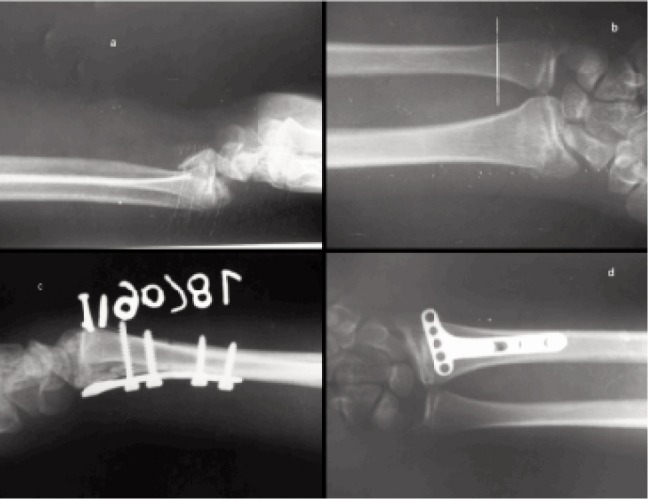
Radiographs a) Lateral view: intra articular distal end radius fracture, b) Antero posterior view: intra articular distal end radius fracture, c) immediate postoperative lateral view showing well reduced intra articular fragment, d) postoperative AP view showing cortical screws only proximal to fracture site, with empty distal screw holes.

**Fig. 2:**
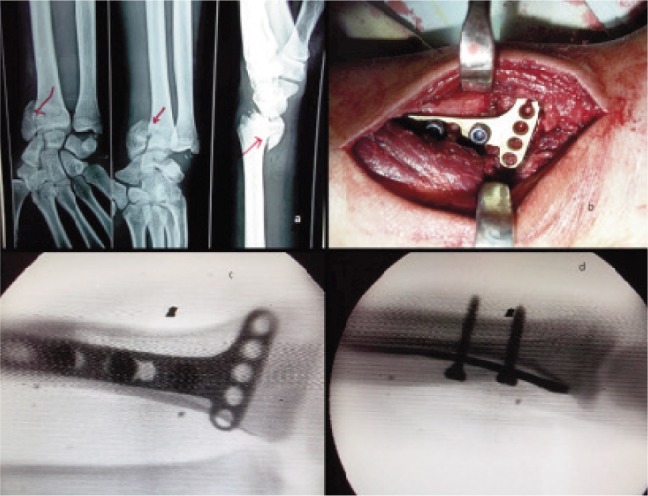
a) preoperative radiographs showing intra articular distal end radius fracture with open physis, b) intra operative radiograph showing screws only proximal to fracture site, c) & d) image intensifier views of reduced fracture using volar buttress plate.

**Fig. 3:**
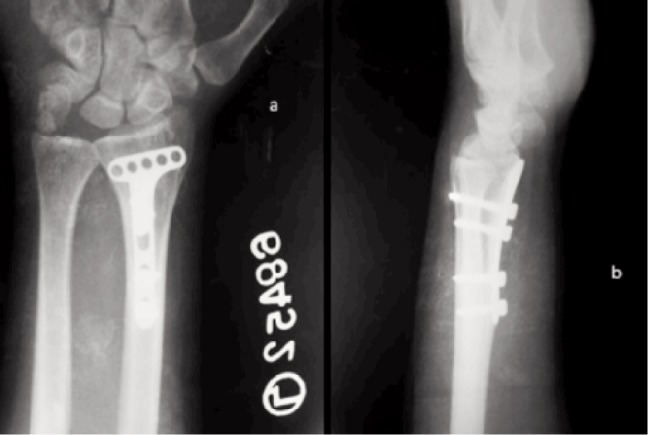
a) United fracture in A-P view with closed radius physis without changes of arthritis at final follow up, b) united intra articular fracture with normal wrist joint at final follow up.

**Fig. 4:**
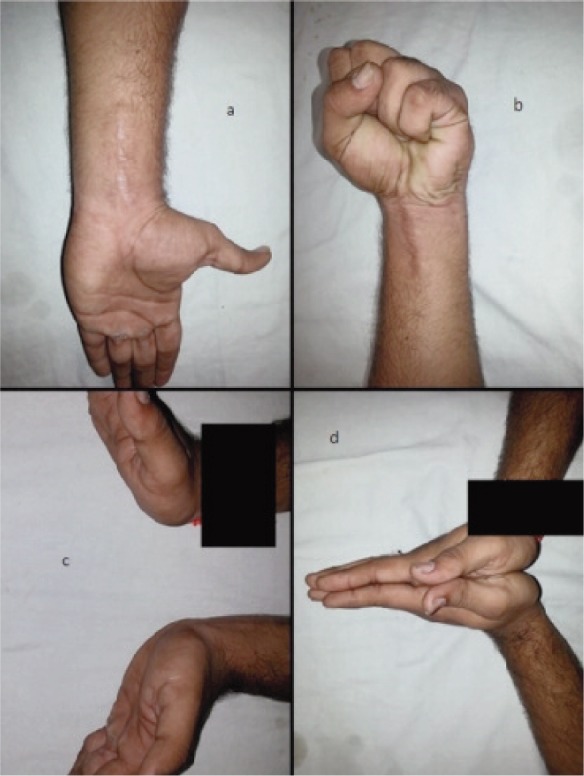
at final follow up: a) well healed surgical wound, b) normal grip, c) normal extension as compared with the other wrist, d) normal extension.
